# The factors affecting the institutionalisation of two policy units in Burkina Faso’s health system: a case study

**DOI:** 10.1186/s12961-017-0228-2

**Published:** 2017-07-17

**Authors:** Andre Zida, John N. Lavis, Nelson K. Sewankambo, Bocar Kouyate, Kaelan Moat

**Affiliations:** 1 Clinical Epidemiology and Biostatistics Unit, Makerere University College of Health Sciences, PO Box 7072, Kampala, Uganda; 2 Ministry of Health, 01 PO Box 7009, Ouagadougou 01, Burkina Faso; 3 McMaster Health Forum, Centre for Health Economics and Policy Analysis, Department of Clinical Epidemiology and Biostatistics, McMaster University, 1280 Main St. West, MML-417, Hamilton, ON L8S 4L6 Canada; 4 Department of Political Science, McMaster University, 1280 Main St. West, MML-417, Hamilton, ON L8S 4L6 Canada

**Keywords:** Institutionalisation, Institutional framework, Data production, Resource availability, Burkina Faso, Policy, Health system, Human resources

## Abstract

**Background:**

This paper is one of three linked studies that attempts to understand the process of institutionalisation of policy units within Burkina Faso’s health system. It examines the relationships between the existence of an institutional framework, data production capacity and other resource availability in the institutionalisation of policy units in health systems. It therefore contributes to our understanding of the dynamics linking the key drivers and indicators of institutionalisation. Additionally, it examines how factors within the managerial setting, including workplace environment, and budgetary and human resource availability, may influence the institutionalisation process.

**Methods:**

The study used an explanatory qualitative case study approach, examining two policy units in Burkina Faso’s Ministry of Health, the first of which had been institutionalised successfully and the other less so. Data were collected from key policymakers, including 13 connected with the first policy unit and 10 with the second, plus two funders. We also conducted a documentary analysis of the National Program for Health Development, two mid-term strategic plans, 230 action plans, eight Ministry of Health state budgets, eight Ministry of Health annual statistics reports, 16 policy unit budgets and published literature.

**Results:**

The framework within which the government gave the policy unit its mandate and policy focus had the strongest effect on the institutionalisation process. Institutionalisation depended on political will, in both the host government and any donors, and the priority given to the policy unit’s focus. It was also affected by the leadership of the policy unit managers. These factors were influenced by human resource capacity, and our findings suggest that, for successful institutionalisation in Burkina Faso’s health system, policy units need to be given sufficient human resources to achieve their objectives.

**Conclusion:**

Policy units’ institutionalisation in Burkina Faso’s health system depend on the leadership of the unit managers to implement relevant activities, mobilise funding, and recruit and maintain enough human resources, as well as the mandate given by the government.

## Background

Over the last few decades, several important policy developments in the health system in Burkina Faso have resulted in significant transformations at the central, regional and district levels. Many of these transformations have resulted in the establishment of new (or the reform of existing) governmental units responsible for overseeing policy and program development and implementation. These policy units include the Directorate General of Infrastructure, Equipment and Maintenance, a mental health unit, and the Permanent Secretariat of the National Program for Health Development. Unfortunately, several of these new policy units have failed to thrive and improve policy development and implementation in the country’s health system [1]. In the latest National Program for Health Development, the government signalled that it will place increased importance on understanding how new policy units within government are organised, particularly as they are seen as key to improved policy implementation [2]. One of the factors often seen as important for success when establishing new policy and program units within government is the extent to which the policy unit becomes institutionalised.

The concept of ‘institutionalisation’ has several connotations and definitions, although all of these have at least one point in common – they agree institutionalisation is an ongoing process in which a set of activities becomes an integral and sustainable part of a formal system [3]. Some scholars [4] have treated institutionalisation as an ongoing process that “*modifies the organization in a stable manner*” so that its elements are fully incorporated into standard practice and used over time [5]. Institutionalisation has also been viewed as a sequence of events leading to “*new practices becoming standard practice*” [6].

The institutionalisation of a specific policy unit may have some specific requirements. For instance, for a policy unit to be considered institutionalised, it must have a virtual or physical ‘head office’ in which it is housed [1]. The institutionalisation of a policy unit can be understood by the extent to which its data and informational outputs are routinely used to inform decision-making processes. In the context of health systems, policy unit institutionalisation has been acknowledged as a fundamental process that ensures core functions continue in a routinised manner across generations of policy unit members (e.g. healthcare professionals, managers, administrators and policymakers), and that these core functions eventually become standard practice [7].

Institutionalisation plays an important role in establishing functional policy units that can help improve health policy development and implementation processes, and ultimately strengthen health systems. The challenges experienced in institutionalising health policy units and programs within the Ministry of Health in Burkina Faso are therefore particularly troublesome, especially because of ongoing concern about poor health indicators in the country. As far as we can gather, no previous studies have identified or explored factors that could support the successful institutionalisation of a policy unit in Burkina Faso.

Several knowledge translation initiatives have been introduced in Burkina Faso to improve evidence-based decision-making, one of which is the rapid response service. This service is provided by a small unit in Burkina Faso’s Ministry of Health, located in Ouagadougou (the country’s capital) and funded by the European Union. Its aim is to provide policymakers with rapid access to appraised research evidence about health systems. The potential benefits to the decision-making process from this service are clear. However, the future of this knowledge translation platform is in the balance, as has commonly been the case with donor-funded projects or programs in Burkina Faso. Their transition into local ownership and their institutionalisation thereafter have often been fragile, and many have collapsed following the end of the donor funding. To support the institutionalisation of this unit, we undertook three separate but linked studies. The first [8] explored the policymaking process leading to the creation of a unit in the health system, and the factors that influenced this process. The study discussed in this paper examines two cases that may provide insights into the factors that facilitate and hinder institutionalisation of policy units in Burkina Faso, to help determine the factors that could aid the rapid response unit in becoming institutionalised and to thrive as a support for decision-makers. The third paper [9] evaluated the process and extent of institutionalisation of the rapid response service itself.

This study therefore aimed to develop a deeper understanding of the specific factors that have facilitated or hindered successful institutionalisation in the health system in Burkina Faso. It focuses on two policy units, the first set up to administer the National Health Accounts Unit (NHAU) and the second established to run a non-communicable diseases program within the Ministry of Health, which has been renamed the Program for Fighting Non-Communicable Diseases (PFNCD) as a pseudonym in this paper to assure its anonymity.

## Methods

An explanatory qualitative case study design was used [10, 11]. Case studies enable the investigator to develop a rich account of complex phenomena that need to be understood within their own context [12, 13]. Case studies are recommended when it is impossible to dissociate a phenomenon from its context [11]. The aim of this study was to consider both the phenomenon (institutionalisation) and its context (the factors in the country that either facilitate or hinder it), so this approach is ideal. The explanatory design was also appropriate given the study’s objective to explain which factors can lead to successful institutionalisation in the Burkina Faso context.

### Study setting and case selection

We chose Burkina Faso as the setting for this study for several reasons. First, the principal researcher has strong professional relationships with key policymakers and stakeholders engaged in the establishment of new health policy units. Second, all the researchers either have experience and knowledge of the policy context in the country, or have worked closely with policymakers and stakeholders who do. This offered the opportunity to develop robust, comprehensive, and verifiable accounts. Third, Burkina Faso is broadly representative of several countries in West Africa, with similar health system structures, population characteristics, and colonial and cultural heritages. Although we did not set out to develop generalisations that could be applied in other settings, the insights derived from this study may prove useful for others in West Africa who are grappling with similar issues.

A case was defined as the process of developing and implementing a policy unit in the Ministry of Health, and the success or failure of institutionalisation of the policy unit during this process. Case selection used purposive sampling [14]. To understand the factors that can lead to successful institutionalisation and those that can hinder this process, we decided to select one policy unit in the Ministry of Health that was generally agreed to have institutionalised successfully, and one whose institutionalisation was considered less successful. The first unit was selected as successful because it has been used as a case study and cited as an example of successful institutionalisation by the World Bank [15, 16]. The second unit was selected using the personal knowledge of one of the authors (BK), who was aware of the problems in that unit because of his professional position.

The two cases selected were the NHAU and the PFNCD. Each policy unit is responsible for the administration, oversight and implementation of a particular strand of health policy in Burkina Faso. They vary in the extent to which they have been successfully implemented and institutionalised, with the NHAU agreed to represent a successful model in the sub-region [1], while the PFNCD still struggles on many levels. At the time of the study, it was not considered an example of successful institutionalisation [1, 2]. These cases provided an opportunity to empirically assess the full range of factors that may explain the differences in the extent of institutionalisation; doing so will equip policymakers and stakeholders in Burkina Faso with an understanding of the elements required to develop and implement a new policy unit in the Ministry of Health. Within the Ministry of Health, it is easy to recognise the people interviewed through the information they have provided, which is why some participants were hesitant to share information, especially about the PFNCD, which was seen as a failed initiative. Therefore, to keep anonymity and confidentiality, the second unit’s name was hidden and replaced by PFNCD.

We studied the progress of each policy unit from January 2005 to January 2016, the date of the interviews. This period was chosen for several reasons. First, it covers both the midway and final evaluation points for the 2001 to 2010 National Program for Health Development, and the development of the new National Program for Health Development 2011–2020. This enabled us to identify problems and the strategies developed to address them. Second, the period also covered the completion, in January 2012, of the health system performance reviews, evaluating health policies and programs in Burkina Faso, but allowed participants to reflect on activities since that time and up to the date of the interviews.

### Study framework

The literature discussing the processes through which institutionalisation occurs is diverse. For example, Yazicroglu and Koc [17] defined institutionalisation as the administration of an organisation in the context of its objectives and targets, but set within a basic philosophy that asserts that it relates to tasks and process models, not individuals. In contrast, Selznick [18] focused on simplicity, differentiation, flexibility and self-determination. Berger and Luckman [19] approached institutionalisation as a social construction of reality, but suggested habituation and standardisation were also important in the institutionalisation process [3, 19, 20]. Many other scholars have approached the concept as part of the life cycle, evolution and sustainability of health programs [21–25].

There is a huge breadth and diversity of analytic frameworks available to better understand institutionalisation. We used one proposed by the World Bank [3] to assess the level of institutionalisation of the two selected policy units. The framework provides three clear categories to focus and organise the analysis of data, namely (1) the existence of an institutional framework; (2) the standardisation of data gathering and reporting systems; and (3) the availability of adequate resources to implement activities. Each category also includes a set of clear indicators to determine the extent to which institutionalisation has occurred (Table 1).

**Table 1:** Indicators of policy unit institutionalisation

Institutionalisation elements	Indicators
1. Existence of an institutional framework (the policy unit’s mandate from government)	• Law/regulation providing a mandate for the policy unit • Institutional home identified for the policy unit • Protocols/public norms set out for data or information production
2. Consistent production of data and preparation of reports	• Explicit process designed for data gathering, compilation and transmission for decision-making • Policy unit activities are regular and ongoing • Protocol exists for validating reports • Minimum set of globally agreed data is produced
3. Adequate financial and human resources, and infrastructure capacity to routinely produce and make use of data in policymaking	• The policy unit has an annual plan of action • Government budget is earmarked for the policy unit’s activities • Sufficient material and human resources are available for the policy unit’s activities • The unit’s annual action plan is at least half funded

Source: based on the World Bank framework [3]

### Data sources

We used several sources of data to ensure robust and comprehensive accounts of each case. We conducted semi-structured interviews with key informants, using open-ended questions developed to explore the different concepts and indicators in our analytic framework. Interviewees were chosen from among those who had worked with or in one of the two policy units studied, and from donor organisations. Non-donor respondents all worked in the Ministry of Health, but at the time of the interview, not all worked within one of the two policy units that were the focus of this study. These respondents were included because they had participated in either setting up or implementing the activities of at least one of the two policy units.

We also conducted an extensive documentary analysis on a range of reports produced by the two policy units, as well as government health system action plans and state budgets from 2005 to 2016. The principal investigator (AZ) acted as a participant-observer, having worked in the health system for several years, including on establishment of the NHAU, and drew on extensive knowledge of the health system in Burkina Faso to add additional detail to each case description. To ensure knowledge about and experiences working in the health system did not detract from the principal investigator’s ability to remain objective throughout the conduct of the study, a second researcher (KM) was consulted from time to time to triangulate initial findings and corroborate the interpretation of our results.

### Data processing and analysis

Completed interviews were transcribed in full and used as the primary data source. Transcriptions were re-read and coded using the themes outlined in the analysis framework (existence of an institutional framework, consistent production of data and preparation of reports, and adequate financial, human resources and infrastructure capacity to routinely produce and make use of data in policymaking). The same coding was applied to the review of documents. Excerpts from both data sources were organised into tables using a word-processing program [26, 27] to organise and merge initial results from both data sources [26]. We compared the data with the indicators and determined the extent to which each policy unit had been institutionalised within the time frame. We characterised the degree of institutionalisation using a four-point scale, where 1 was very unsatisfactory, 2 unsatisfactory, 3 satisfactory and 4 very satisfactory. Finally, we used a comparative synthesis to identify the factors that underpinned successful (i.e. satisfactory or very satisfactory) or unsuccessful (i.e. unsatisfactory or very unsatisfactory) institutionalisation. To ensure consistency in the analysis, two of the authors (BK and KM), both of whom were knowledgeable about the units concerned but not involved in the data collection, reviewed the study’s major findings for coherence.

## Results

A total of 25 people were interviewed, including 13 connected with the NHAU, 10 with the PFNCD, and two from donor organisations. Documents reviewed included the National Program for Health Development, two mid-term strategic plans, 230 action plans, eight Ministry of Health state budgets, eight Ministry of Health annual statistics reports, and 16 state budgets and articles.

### Case 1: The NHAU

The NHAU provided a systematic approach to mapping the annual flow of health system funds around the health system in Burkina Faso [28]. The process involved bringing together data from a variety of sources, including the public sector, donors and private sector, and undertaking an analysis of health system expenditure. Reports were designed to support policymaking in a user-friendly way [29].

The setting up of this policy unit was included in the National Program for Health Development and in each annual action plan published by the Ministry of Health since then, reflecting the importance placed on this unit by both the Ministry and donors. Burkina Faso benefited more generally from this initiative because it facilitated capacity-building among those responsible for the work. Those involved in preparing the reports built technical capacity to use the methods needed to apply the National Health Accounts (NHA) framework, establishing a technical team able to present relevant quality indicators underpinned by recent data. As a result, Burkina Faso was cited as a model for regular NHA data production in West Africa [3].

The NHA had been produced since 2005 without a formal policy unit, and had contributed significantly to the work of organisations making policy on health financing in Burkina Faso. For example, Amnesty International used NHA data from Burkina Faso as part of its reproductive health campaign in the country, calling on the government to expand and improve access to family planning services, remove financial barriers to maternal healthcare services, and ensure an even distribution of health facilities and trained staff across the country [30, 31]. More recently, the International Labour Organization used NHA data to test the feasibility of a social health insurance program [32, 33]. The policy unit was established in the Ministry of Health in 2009, with a mandate to produce the NHA data every year [34].

#### Existence of an institutional framework

The establishment of the NHAU was a shared commitment by the government and donors, mainly because of its perceived added value as an input into decision-making about health financing within the Ministry of Health. Its establishment, however, was proposed by donors, particularly those aligned with WHO, and the government then took ownership. Several participants mentioned that donors were the key support for the NHA studies in Burkina Faso, with one summing it up particularly well as:

“*…after the presentation of the first national health accounts results in 2004, many policy actors at the Ministry of Health appreciated the results and the methodology used. We continued the process with World Health Organization support. …without World Health Organization financial and technical support, Burkina Faso would not have been able to do the health accounts because of the cost and we did not have the technical expertise…*” (Project coordinator)

The NHAU was placed in the department of planning of the Ministry of Health, before moving to the department of health information and statistics, where it has resided since [35]. The policy unit was not established by a unit-specific decree. Instead, it was established within a more general legal text produced by an inter-ministerial decree signed in 2007 to create steering and technical committees to define guidelines. This legal text outlined the responsibility for the functioning of the NHAU, and clearly defined its human resources need, and the role of each individual in the process.

#### Consistent production of data and preparation of reports

The NHAU data and reporting system was supported by the existence of a health accounts framework and tools. This supported the adaptation of the health accounts to rapidly-evolving health financing systems, further enhanced within- and between-country comparability of health expenditure and financing data, and ultimately improved the information base for the analytical use of the data. One participant noted:

“*…the system of health accounts (SHA-2011) framework and the health accounts production tool were useful to understand the health financing data mapping and analysis for many people. Before that, Burkina Faso was using Microsoft Excel for the data analysis and it was difficult to understand and transparency was not clear …*” (Health accounts steering committee member)

The framework also reflected the complex and changing systems of healthcare financing and eliminated ambiguities around some of the concepts related to financing. It allowed low- and middle-income countries like Burkina Faso to provide a more transparent picture of donor assistance.

WHO and the United States Agency for International Development (USAID) have developed two tools to streamline production of health accounts [36], which are used to facilitate the process of producing data and generating reports in Burkina Faso. The first is the Health Accounts Production Tool, which provides step-by-step guidance to users, and the second is the Health Accounts Analysis Tool, which supports data analysis.

By the end of the study period, the NHAU in Burkina Faso no longer collected any public data. The most important data were instead collected through an established health expenditure database, although the process was not fully integrated into routine data collection within the health information system. Burkina Faso was regarded as the most advanced country in West Africa for health account data production and was now supporting others, as one participant noted:

“*…Burkina Faso is now a reference in Africa for health accounts. Since 2003, the country has consistently produced health accounts… we have more than 15 health accounts studies while some countries are struggle to complete one. Some of health accounts team members are now assisting other countries to apply the new health accounts framework and to use the health accounts production tool… members of Burkina Faso’s steering committee have assisted more than 15 countries in Africa, Asia and Caribbean America …*” (Health accounts steering committee member)

Since its start up, the NHAU had been characterised by a steady production of accounts under the coordination of the executive director. Since 2005, there had been 10 NHA and 15 sub-accounts produced (a sub-account provides systematic, comprehensive and consistent monitoring of resource flows in a health system for a specific disease or health program) [37].

#### Adequate financial, human and infrastructure capacity to routinely produce and use health accounts

##### Financing

The NHAU was funded from two main sources, the state budget (for salaries) and donors. The state funds were intended to support salary increases for employees and infrastructural demands (e.g. technical equipment and materials required to undertake each NHA). However, the government’s contribution was low and often insufficient to support the ongoing operation of the policy unit. The NHAU did not have a specific state budget line for data collection, processing and analysis. One participant noted:

“*…to be independent, the health accounts unit needs its own budget line to collect and analyse data every year. Donors are the main source of funding to produce the health accounts every year. What we have as a state budget is not for the health accounts unit but for the entire directorate, although we use it for some operating costs like printing, telephone and sometimes gas.*” (Health accounts steering committee member)

Donors therefore provided most of the funding, supporting ongoing data collection, processing and analysis, as well as the preparation of reports and dissemination of the results. Since 2005, the main donors had been WHO, the World Bank, the Luxembourg Project and the Global Fund. The NHAU also received financial support from the Japanese Cooperation for International Development in its early years of operation. As is clear from the earlier quote, the difficulties related to ensuring a sustainable source of financing for the NHAU were linked to the absence of a state budget line. The policy unit was considered vulnerable should any of the current donors withdraw support [12].

##### Human resources

Study respondents suggested that the NHAU was understaffed, with only two individuals working full time and 16 part-time. The small team included personnel from a number of disciplines, including health economics and statistics, all of whom were trained by the WHO and the World Bank. To strengthen their capacity, continuous training was provided to all staff, including sessions at the national level as well as specialisation courses such as a mix of national and regional workshops.

The NHAU had operational autonomy under the supervision of technical and general directors. There were no individual-level descriptions of positions or duties, but the inter-ministerial decree described stakeholders’ roles in the annual process of preparing the accounts.

##### Infrastructure and equipment

Infrastructure and equipment were one of the major constraints on the NHAU’s operation. The policy unit had a head office but was characterised by inadequate infrastructure and equipment, including computers and related equipment (e.g. printers). Several respondents noted that, despite having new computers, the ongoing maintenance required to keep them fully functioning was not provided and equipment was seldom upgraded. Some respondents said that this was a particular challenge, citing equipment funded by WHO in 2010, which had not been upgraded or maintained appropriately. One health accounts steering committee member commented:

“*…I am working with my own laptop and as you know we have not had internet connection in this office for many years and nobody cares about it…I hear that WHO provided laptops and a server in 2010 but I don’t think all of them are still working, … We have enough space but the concern is internet and our electricity is not permanent…*” (Health accounts steering committee member)

These responses suggest the infrastructure and equipment dimension of institutionalisation was one of the major weaknesses in NHAU institutionalisation, although it did not significantly impede the functioning of the policy unit. Overall, our analysis suggests the main elements contributing to the institutionalisation of the NHAU were:

1. A clear mandate from the government through an inter-ministerial decree;
2. Regular production of data (in consistent annual reports) to support policymaking; and
3. Funding commitments by donors, although this may also be viewed as a challenge given the potential for donor withdrawal.

### Case 2: The PFNCD

The PFNCD aimed to provide medical care to people suffering from non-communicable diseases. The policy unit operated under the Directorate for the Control of Neglected Tropical Diseases in Burkina Faso’s Ministry of Health. It was established by the Ministry of Health in response to WHO’s suggestion that the incidence of many non-communicable diseases could be reduced by designed and targeted programs at the national and international level [37]. The establishment of the policy unit was supported by other units within the Ministry of Health (including the Departments of Planning, Directorate of Disease Control and the Directorate General of Health). The initiative also included new screening programs for early disease detection, awareness-raising about the risk factors and symptoms associated with the development of non-communicable diseases, and extended medical care for people suffering from a range of non-communicable diseases. Though professionals were providing care for those suffering, in many areas in Burkina Faso people were unable to access care for many reasons, including lack of funding to expand services in all provinces, sociocultural barriers, and illiteracy [38].

#### Existence of an institutional framework

The PFNCD was established because the government wanted a way to address non-communicable diseases in the National Program for Health Development. Non-communicable diseases were recognised as having been given very little consideration in the process of policy planning and health system financing, which had led to difficulties in implementing activities to reduce their burden on the nation’s health. One participant commented:

“*…non-communicable diseases are known as most of the poor health programs in Burkina Faso’s health system; state budget lines are insufficient to implement our activities… among the donors there are few interested in putting their money into our programs… the lack of financial resources makes it difficult to maintain human resources for long because it is difficult to implement program activities…*” (Senior policymaker)

The PFNCD had been incorporated into the Ministry of Health’s organisational chart as part of the Directorate of Disease Control. These structures suggest a strong institutional framework exists to support the PFNCD, which could be seen as a facilitator of successful institutionalisation.

#### Consistent production of data and preparation of reports

The policy unit faced some challenges in data production, including weak data sources and shortcomings in data management, collection and processing. There were also clear weaknesses in the policy unit’s ability to produce information for policy and planning purposes, underpinned by a lack of human resources with the necessary reporting skills.

“*…if you look in the annual health statistical report there are few indicators for non-communicable disease…if you also look at the progress report (a list of indicators requested by the basket fund unit), since 2007 there are no indicators for our health program…for sure the health districts are getting the basket fund money but not mainly for activities related to non-communicable diseases… with this lack of funds it is difficult to collect our own data or to conduct studies or research related to our health program.*” (Project coordinator)

Our analysis suggests that the coordination, planning and leadership of the policy unit could be considered a weakness. The policy unit had no strategic or operational plan, internal coordination mechanisms or systems for coordination with other actors in the health system. The policy unit had also not proposed any indicators for collection and did not participate in health information system activities. There were no initiatives planned to make any improvements in this domain in the near future.

#### Adequate financial, human and infrastructure capacity to routinely produce and use health accounts

##### Financing

Unlike the NHAU, the state budget was the main source of financing for the PFNCD unit, providing a salary for the program coordinator. According to one of the respondents:

“… *the last funds received from a donor were seven years ago, in 2006; that is why nobody wants to come and work in this program.*” (Worker on one non-communicable disease program)

Despite having a dedicated budget line, the PFNCD shared a budget of 100 million FCFA (approximately 200,000 USD) with all other non-communicable disease programs. The PFNCD’s share of the annual budget was approximately 5 million FCFA (equivalent to 10,000 USD), which several respondents noted was not enough for any operational activity, and no activities were planned for fundraising. Respondents also suggested that, as a result of the lack of funding, the policy unit appeared not to function, and was clearly seen as not being a government priority. For example, one said:

“*…due to the lack of funds, the PFNCD is being implemented by one person. At activities planning and funding meetings, there is usually no representative from the program side to defend the activities and get them funded….as you can see, this office houses four non-communicable diseases programs, but the PFNCD has nobody. The program seems not to be functioning and no health workers are willing to work there because of the lack of funding to implement the activities*…” (Worker on a non-communicable disease program)

##### Human resources

Analysis of the human resources associated with the PFNCD also highlighted shortages. Official figures showed only one person in the policy unit, who was also the only specialist in the entire country for the medical care of patients suffering from diseases covered by the program [39]. The coordinator was the only person in the policy unit, which resulted in administrative and procedural blockages, mostly related to this person’s consistent non-availability. There may have been opportunities to delegate some work to junior staff, but the program was characterised by the absence of plans or processes to guide the delegation of authority, meaning nearly all decisions were still taken by a single person.

##### Infrastructure and equipment

The PFNCD had very little infrastructure or equipment. Respondents reported that the policy unit shared a single office with three other programs, and did not have additional space for any staff that may be recruited into the unit in the future. One respondent said:

“*All the program equipment is in the office of the coordinator, how do you expect the policy unit to work?*” (Worker on one non-communicable disease program)

It is therefore clear that the PFNCD faced several challenges to institutionalisation. While the policy unit had been mandated by the government (an institutional framework), it was not producing any information to inform policymaking, had not established clear indicators within the health information system, so could not collect any data, and had a shortage of financial and human resources to plan and implement activities.

### What leads to successful institutionalisation: comparing cases

Our analysis has shown that one policy unit (NHAU) had experienced a relatively successful path to institutionalisation, while the other (PFNCD) had failed to institutionalise successfully. This difference can be explained by looking at the existence of an institutional framework, the consistency with which reports to support decision-making were produced, and the extent to which financial and human resources and infrastructure could support operations.

#### Existence of an institutional framework

There were many similarities in institutional frameworks between the two policy units. The aim of an institutional framework is to ensure that there is a regulatory framework through which a policy unit is authorised to exist and function. It is important in establishing clearly-defined roles and responsibilities of all stakeholders. Our comparative analysis (Table 2) showed that there were similarities between the two cases. First, both had government mandates and were underpinned by satisfactory regulatory frameworks. Second, both were integrated into the ministerial organisational chart, with the NHAU in the Department of Health Information and Statistics, and the PFNCD under the Directorate for Fighting Non-Communicable Diseases. Third, both had a home in the Ministry of Health, although both experienced office space and equipment constraints. Fourth, both policy units had established protocols and norms to implement their activities, with the NHAU using the system of health accounts framework (SHA-2011) and the PFNCD using an established care and treatment protocol.

**Table 2:** Comparative analysis of institutionalisation indicators in the two policy units studied

Institutionalisation elements	Indicators	Units	
		National Health Accounts Unit (NHAU)	Program for Fighting Non-Communicable Diseases (PFNCD)
1. Existence of an institutional framework (the unit’s mandate from government)	1.1. Law/regulation providing a mandate for the unit	➢ There is an inter-ministerial decree signed by the ministers of health and economy and finance; this identifies the stakeholders and their role in the production of health accounts ➢ An integrated organisation chart is aligned with the organisation chart for the overarching directorate	➢ There is an institutional framework that allows the implementation of the program ➢ There is an integrated organisation chart aligned with the organisation chart of the service for the fight against non-communicable diseases
	1.2. Institutional home identified for the policy units	➢ There is a dedicated working space for this unit but it is insufficient for all staff	➢ There is working space available in an office, but shared with four other policy units
	1.3. Protocols/public norms set out for data or information production	➢ WHO’s system of health accounts framework (currently SHA-2011) is the main guideline for health accounts	➢ There are protocols for medical care amongst those with these diseases
2. Consistent production of data and preparation of reports	2.1. Explicit process designed for data gathering, compilation and transmission for decision-making	➢ There is no system for routine gathering of health expenditure in the health information system ➢ The service uses a database of health expenditure developed in 2008 and integrated into the health system ➢ A survey is carried out each year, funded by donors, to provide additional data	➢ There is no data collection system, unlike other policy units for other diseases, although the unit has a data collection guideline ➢ No routine data are collected and no indicator from the unit is included in the statistical yearbook, the reference for data gathering and use in policymaking
	2.2. Policy unit activities are regular and ongoing	➢ Ten sets of health accounts, including sub-accounts, have been produced since 2005 ➢ Health accounts are poorly used in decision-making processes	➢ No policy unit activity was documented
	2.3. Protocol exists for validating reports	➢ Data collection is not integrated into routine data collection processes, but the policy unit uses a standard health expenditure database	➢ There is a lack of coordination among stakeholders, which hinders data production ➢ There is a lack of involvement of stakeholders (NGOs, municipalities, social services) in caring for patients with these diseases
	2.4. Minimum set of globally agreed data is produced	➢ Health system expenditure data are available every year ➢ Data are processed using the health accounts production tool and the health account analysis tool provided by the WHO and US Agency for International Development	➢ No administrative reports, evaluation reports or other publications have been developed and published
3. Adequate financial and human resources, and infrastructure capacity to routinely produce and make use of data in policymaking	3.1. The policy unit has an annual action plan	➢ There is an action plan for the health information and statistics department which includes the NHAU’s activities	➢ There is an action plan for the overarching Ministry of Health department, which should include PFNCD activities, but little is planned
	3.2. Government budget is earmarked for the policy unit’s activities	➢ There is no budget line from the Ministry of Health ➢ The state budget contribution is mainly for salaries ➢ The unit’s activities are typically funded by donors; there is usually no difficulty obtaining the money but there may be delays in releasing funds	➢ There is a state budget line covering four health programs; the portion allocated to the policy unit is insufficient ➢ There are no donor funds available; the last such support was in 2006
	3.3. Sufficient material and human resources are available for the policy unit’s activities	➢ The policy unit obtained computer equipment in 2010; it uses hardware from the health information and statistics unit ➢ There are sufficient multidisciplinary resources from several departments; however, staff must be regularly trained and financially motivated to retain them	➢ The PFNCD does not have enough equipment; it lacks computers and office furniture ➢ The PFNCD has just one member of staff, the policy unit coordinator, who is rarely available and mostly absent during planning, which hinders the mobilisation of resources
	3.4. The policy unit’s annual action plan is at least half funded	➢ The history of the policy unit’s annual action plan shows that, since 2009, at least more than 90% of the activities were funded	➢ The records of the policy unit action plans from 2005 to 2012 shows that the unit activities funding rate are between 10% and 16%

#### Consistent production of data and preparation of reports

There were many differences in the extent to which each policy unit engaged in the consistent production of data and the preparation of reports to support decision-making, which require a system that allows routine collection and analysis of established indicators. The NHAU had a clearly-defined and well-understood routine for data-gathering and report production. The data-gathering process was not yet integrated into the health information system, but it used a database housed in the Ministry of Health, drawing on health expenditure data that were routinely collected, validated and institutionalised as of December 2008. Donors’ funds were used to support additional data collection each year. The team used Excel for processing and data analysis purposes from 2005 until 2011, and then two tools provided by WHO and USAID (Health Accounts Production Tool, Health Accounts Analysis Tool) for the production of health accounts. The data produced by the policy unit are supposed to inform decision-making in health financing, although respondents felt that the data were being used poorly for this purpose, with one saying:

“*…like all other research studies the health accounts results are poorly used for policymaking. We feel like donors are using the health accounts results more than the policymakers at the ministry of health. …the health account team know how to analyse the health accounts data, they have good experience, they should now learn how to push the results to be used for policymaking…*” (Senior policymaker)

The PFNCD, however, did not lead any routine data collection and analysis activity, and had no clearly-established indicators that the government had prioritised for routine reporting. Unlike the NHAU, the PFNCD had no established processes or guidelines for data management. No regular reports were produced, so there were no opportunities for clear linkages between the policy unit and decision-making processes. Study respondents suggested that there is a relationship between human resource capacity and the establishment of routine data collection, management and reporting processes.

#### Adequate financial, human resources and infrastructure capacity to routinely produce and make use of data in policymaking

The NHAU had two main sources of funding, namely donors that financed data collection, maintenance, analysis, reporting and results dissemination, and government resources from the state budget, which covered employees’ salaries. Some funding was allocated from the state budget to the PFNCD, but this was inadequate to support day-to-day operations, and no donor funds had been allocated. The PFNCD therefore had very little funding, which made it harder to attract good staff. From 2005 to 2012, the total amount allocated to NHAU (505,000 USD) was almost 10 times that allocated to PFNCD (55,900 USD).

Our results showed that while financial and human resources and infrastructure were moderately satisfactory for NHAU, this was not the case for the PFNCD. Having two full-time and 16 part-time employees in the NHAU still left the policy unit facing significant challenges, but this was in stark contrast to the single person working in the PFNCD. The NHAU had a multidisciplinary team all trained to use the NHA framework, with support from the WHO and the World Bank. Some respondents highlighted dissatisfaction among those working in the NHAU, often the result of a lack of motivation, but each team member had a clearly defined role. The PFNCD had no autonomy nor job descriptions.

Equipment, and specifically the ongoing maintenance and upgrading of computing equipment, remained one of the constraints inhibiting the smooth operationalisation of the NHAU. It was, however, still much better equipped than the PFNCD, which suffered from a lack of both infrastructure and equipment. The NHAU had a head office with space for all employees, but the PFNCD was located in an office shared by three other programs, and did not even have desk space for any staff that may be recruited into the policy unit in the future.

#### Factors associated with successful institutionalisation

This study has identified a number of factors contributing to the successful or unsuccessful institutionalisation of a policy unit in the Burkina Faso health system (Table 3). There are necessary preconditions for successful policy unit institutionalisation in Burkina Faso’s Ministry of Health. We suggest that the existence of an institutional framework is a necessary but not sufficient condition, because both cases studied had an established framework but experienced varying degrees of institutionalisation success. Adequate financial and human resources are also key. Giving the policy unit an official mandate, with a clear policy issue or health problem to address, is vital in the institutionalisation process, but as shown by the case of PFNCD, clearly not enough on its own. The adaptation of the World Bank framework for this study is useful for guiding the analysis of a policy unit’s institutionalisation and also highlighting institutionalisation facilitators and barriers elements. Table 3 below outlines factors affecting policy unit institutionalisation that could be incorporated into the institutionalisation process to mitigate risks.

**Table 3:** Factors affecting institutionalisation

Institutionalisation elements	Facilitators and barriers
Existence of an institutional framework (the unit’s mandate from government)	• Environment and governance structure: Creating an official decree or mandate to ensure that the unit operates under favourable conditions. Institutional arrangements and coordination within and between internal and external stakeholders is also necessary • Aspects of unit strategy and features: These include the nature of the design process and the start-up, particular whether local stakeholders were involved in the unit’s establishment, whether the unit meets stakeholders’ needs and fits the environment, whether there is evaluation and how long the unit will remain in place • Managerial factors: These include whether there is a clear policy and unit champion strategically placed to support ongoing implementation, whether the unit’s policy is consistent with the mission and operating procedures of the Ministry of Health, and whether the government is likely to support the unit after any pilot stage • Workplace environment factors: These include the stability and favourability of external socioeconomic and political factors, such as legislation and policy positions affecting the unit (for example, the recent crisis in Burkina Faso affected many health units and their work plans) • Policy implementation factors: These include government and key stakeholders’ views on the level of priority of the unit’s work, and the opportunity cost
Consistent production of data and preparation of reports	• Lack of norms, procedures and functional coordination of the unit • Lack of strategic and operational plan for short- and long-term work • Low human resource capacity for data collection and processing • Weakness and often lack of internet connection at central, regional and provincial level; no functional website and frequent • Data collection tool not adapted to the unit’s needs • Lack of indicators and tools for routine data collection • Lack of processing tools and mechanism for data sharing • Lack of dissemination of information produced by the unit
Adequate financial, human resources and infrastructure capacity to routinely produce and make use of data in policymaking	• Lack of state budget line to support the unit’s activities and ensure continuation • Lack of multiple financial sources (donors, public and private) to support activities • Lack of mid- and long-term planning for resource mobilisation • Sufficient well-trained staff to support unit activities • Infrastructure and equipment availability and good internet connection for data production and information-sharing • Lack of training of local decision-makers, opinion leaders, the media, etc. on how to use information produced by unit for decision-making

## Discussion

### Principal findings

We found that the NHAU was on the path towards institutionalisation in Burkina Faso. We were able to identify most of the criteria in our analytic framework that signal successful institutionalisation, although it was also clear that enhancements to human, financial and material resources were required. In contrast, the PFNCD may be considered one of the most neglected health programs in Burkina Faso, and our analysis suggests that it has not been institutionalised successfully.

Our results also suggest that the institutionalisation of any policy unit depends heavily on the political will of both the state and donors, as this can lead to concrete commitments to stable sources of funding, adequate allocation of human resources and investment in infrastructure and equipment. Another important dimension appears to be the priority given to the policy domain covered by that policy unit, and the leadership demonstrated by the unit managers in proposing activities that may raise additional funds. Our results suggested that these additional facilitating factors are probably underpinned by human resource capacity, which can also support the proposal of new activities, as well as the successful implementation of agreed courses of action. These can both help establish policy unit visibility and credibility, and improve the likelihood that the unit will generate further operational support in both symbolic and real terms. This aligns with previous work [40], which suggested that “*humans are* [the] *greatest assets of organisations, without them, everyday business functions could not be completed*”. Our study suggests that, for successful policy unit institutionalisation in Burkina Faso’s health system, policymakers should ensure that the policy unit has sufficient human resources to fulfil its purpose effectively.

We also found that the government’s perceptions of whether and how a policy unit is achieving its purpose are very important in supporting successful policy unit institutionalisation. Policymakers need to be satisfied with what the policy unit provides, and its activities need to fit within the broader public sector. This also requires investment in modern information management systems for decision-making.

### Strengths and weaknesses of the study

This study had two main strengths. First, following a qualitative case study method [41], our case studies drew on a wide range of sources of data that provided greater depth than could have been obtained through a single data source. This enabled the development of comprehensive accounts of each of the policy units studied. The second strength was the principal investigator’s (AZ) role as a participant-observer in the process. This provided an opportunity to observe and understand many aspects of the case that would not be possible for other investigators, including non-verbal expressions of feeling, determinations of who interacts with whom in the health system, and how participants’ responses are influenced by political context. However, this could also have been considered a weakness, because being a participant-observer reduces objectivity. This might have been a particular issue because the principal investigator had only been involved in establishing one of the two units studied. Two steps were therefore taken to ensure the analysis and interpretation of results related to both policy units were treated in an objective way. Firstly, a second researcher (KM) was involved in and consulted during various stages of data analysis (and in interpreting the results) and, secondly, external reviewers (BK and KM) who were familiar with the health system, political context in Burkina Faso and health policy analysis reviewed the study’s major findings to ensure they were clear, consistent and valid.

This study also had some other weaknesses. First, it was limited to two cases to ensure the study scope was feasible. There are a range of projects and programs in the Ministry of Health in Burkina Faso that illustrate institutionalisation successes or highlight potential problems. These have not been examined, even though they could also provide important information about the factors that can help ensure successful institutionalisation. Second, we interviewed a relatively small number of people for each case, specifically 13 people for NHAU and 10 for PFNCD. However, several scholars have shown that, when studying institutionalisation, there is no need to interview a large number of people, with some authors suggesting that three [42] or even two [43, 44] respondents per policy unit is sufficient for reliability. Considering only the opinion(s) of the managers from the policy units should be avoided to eliminate a positive bias to the assessment [22, 23], which is why our study also included several members of the unit outside the core management team. Third, some participants were hesitant to share information, especially about the PFNCD, which was seen as a failed initiative. That is why the second unit name is hidden and replaced by PFNCD. The lack of data and the need to anonymise the PFNCD limited the scope of certain aspects of the analysis, including policy unit interventions and stakeholder implications. Some interviewees also did not completely understand the concept of institutionalisation and its determining factors. This created some difficulty in obtaining some of the information necessary, particularly if a lack of understanding resulted in a refusal to share important information. Fourth, more detailed analysis should have been undertaken to examine the type of leadership of a policy unit that may lead to its institutionalisation.

### Findings in relation to other studies

Institutionalisation of policy units and programs can be difficult [45]. This is mainly because the levels of uncertainty are high, especially when there is a lack of local ownership and thus lack of consensus on resources (financial, human, material). Problems with institutionalisation also appear when the usefulness and value of the project or program is not recognised at all levels in the health system. Recent studies have found that a lack of evidence-based policymaking, poor availability of underlying data, weaknesses in data-generating systems and linkages to public expenditure management (especially in a decentralised context), plus difficulties in tracking private health expenditure flows are among the main reasons why progress toward institutionalisation of NHA policy units is slowed [46–49]. These factors ultimately indicate a lack of will by policymakers to continue with the policy unit’s activities. The lack of standardised methodologies, tools to estimate healthcare expenditure by NHA units in general and by disease, and inconsistencies between the figures reported by health programs have also hampered institutionalisation. Our findings are clearly consistent with these results.

At present, few studies have been conducted in Burkina Faso to assess the factors affecting the institutionalisation of policy units other than those associated with NHA, so it is unclear whether our results are consistent with experiences elsewhere. A study by Kasonde in Zambia on creating a knowledge transfer platform in the Zambian health system, however, found that the Ministry of Health was becoming a routine demander of the unit’s services. Uncertainty over its funding base remained a persistent feature in whether it was likely to become institutionalised [50], which is consistent with our findings. Our findings are also consistent with a study conducted by El-Jardali et al. [51] on understanding factors that can help to ensure the sustainability of knowledge translation platforms. Three major challenges were raised – first, the location of the platforms; second, ensuring the sustainability after the end of funding; and third, building capacity.

## Conclusions

### Implications for policy and practice

Our findings suggest that political will is a key factor associated with successful institutionalisation. There is, therefore, a clear role for politicians to play in the successful institutionalisation of policy units. This suggests that policymakers may need to be challenged to take a more proactive role in health policy development by ensuring the needs of the different policy units are satisfied, that coordination and harmonisation of the interventions in these units is achieved, and that modern systems of information management are implemented to support their work. The broad consensus is that demand for institutionalisation should be initiated at the national level by senior policymakers, and that it requires the right conditions, including effective parliamentary scrutiny, and an active civil society. Donors also have a role to play in enabling countries to achieve institutionalisation of policy units. Policymakers and donors working together should develop viable solutions to ensure permanent funding for the operation of the policy units. Other key stakeholders, including managers and analysts in the health system, should actively raise awareness of the resources and capacity required to ensure the ongoing operation of an established unit.

### Implications for future research

This research has explored the factors that affect the institutionalisation of policy units in Burkina Faso’s health system. It is based on two case studies, one an institutionalised policy unit and a second that has not been institutionalised. The study examined the relationship between successful institutionalisation and the existence of an institutional framework, consistent production of data and preparation of reports, adequate financial and human resources, and infrastructure capacity to routinely produce and make use of data in policymaking. It therefore contributes to our understanding of the dynamics linking institutionalisation to these specific indicators. The conceptual approach and methodological processes used might be enhanced and advanced in future research. For instance, several other aspects of institutionalisation could also be explored, including the influence of factors related to the political environment, governance, policy unit strategic plans and structural features, individual characteristics of key leaders and the environment. This would complement the findings of this study.

The results from this study should lay the groundwork for future research on the institutionalisation of policy units within the Ministry of Health in Burkina Faso, as well as in other settings. Our methodology could be replicated or triangulated to provide a framework for studying institutionalisation in other policy units in Burkina Faso, and in particular to avoid the major barriers to successful institutionalisation. Future research should consider the long-term mandate on data production, the use of such data for decision-making and involvement of all the stakeholders in the process of institutionalisation. Anonymisation constitutes one of the key issues in our study to maintain the privacy of one of the policy units in particular. Future research may also consider the use of pseudonymisation techniques to design an adequate anonymisation process in a given context. Although pseudonymisation is not recognised as a method of anonymisation, it can be helpful in preserving privacy [52].

## Abbreviations

NHA: National Health Accounts
NHAU: National Health Accounts Unit
PFNCD: Program for Fighting Non-Communicable Diseases
USAID: United States Agency for International Development
WHO: World Health Organization
